# Frog body size responses to precipitation shift from resource‐driven to desiccation‐resistant as temperatures warm

**DOI:** 10.1002/ece3.9589

**Published:** 2022-12-12

**Authors:** Jennifer A. Sheridan, Chase D. Mendenhall, Paul Yambun

**Affiliations:** ^1^ Carnegie Museum of Natural History Pittsburgh Pennsylvania USA; ^2^ Section of Birds Carnegie Museum of Natural History Pittsburgh Pennsylvania USA; ^3^ Sabah Parks Kota Kinabalu Sabah Malaysia

**Keywords:** anurans, body size, climate change, precipitation, tropics

## Abstract

Climate change threatens biodiversity in a range of ways, including changing animal body sizes. Despite numerous examples of size declines related to increasing temperatures, patterns of size change are not universal, suggesting that one or more primary mechanisms impacting size change are unknown. Precipitation is likely to influence the size different from and in conjunction with changes in temperature, yet tests of the interaction between these variables are rare. In this study, we show that a crossover interaction between temperature and precipitation impacts the body size of frogs as the climate warms. Using more than 3000 museum frog specimens from Borneo and climate records spanning more than 100 years, we found that frogs are larger in wet conditions than in dry conditions at cool temperatures, suggesting that resource availability determines body size at colder temperature. Conversely, frogs are larger in dry conditions than in wet conditions at warm temperatures, resulting in a crossover to desiccation resistance as the main determinant of body size as climates warm. Our results demonstrate that global warming can alter the impact of precipitation on life‐history traits. We suggest that increased attention be paid to such interactive effects of climate variables, to identify complex mechanisms driving climate‐induced size changes.

## Graphical abstract

Using more than 3000 museum specimens of 18 frog species from Borneo, collected over more than 100 years, we demonstrate that at cool temperatures, frogs are larger in wet conditions than in dry conditions, suggesting that resource availability drives body size. However, at warm temperatures, frogs are larger in dry conditions than in wet conditions, suggesting that desiccation resistance drives size. This work provides evidence that global warming can alter the impact of precipitation on life‐history traits.

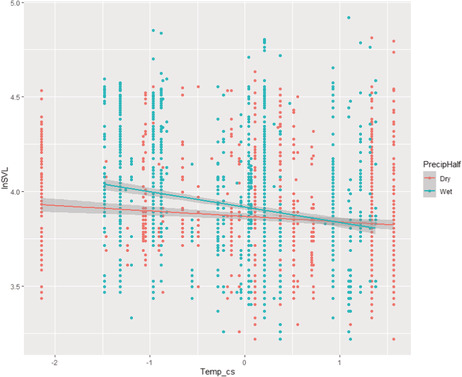

## 1 INTRODUCTION

Temperature and precipitation play a major role in the distribution, physiology, and body size of animals worldwide (Gardner et al., 2011; Parmesan & Yohe, 2003). An increasing number of studies report patterns of pronounced changes in body size across taxa in aquatic and terrestrial environments occurring in the last 50 to 100 years (Sheridan & Bickford, 2011; Weeks et al., 2020), attributed to the impacts of increased temperatures on size. Exact mechanisms for these changes are still debated, but experimental work and broadly observed geographic trends suggest that increased temperatures associated with climate change can reduce body sizes of both endotherms and ectotherms through different pathways.

For endotherms, Bergmann's Rule states that individuals and species are comparatively larger in colder environments to decrease the ratio of surface area to volume for efficient heat conservation (Bergmann, 1847). In ectotherms, the temperature‐size rule predicts that developmental rate increases with temperature while growth rate is less affected, resulting in smaller sizes at first reproduction (Atkinson, 1996). Additionally, because ectothermic metabolism and temperature are linked, individuals and species are comparatively smaller in warmer environments (higher metabolism results in higher resource requirements to achieve a given size; Gillooly et al., 2001). Regardless of the driving factors, expected size declines may lead to population declines and potentially to species losses because reproductive output and survival are often tightly correlated with size (Roff, 2002). Thus, size declines may be a harbinger of future population declines, exacerbating the current trend of nearly one‐third of known vertebrates declining in population size and range (Ceballos et al., 2017).

Despite numerous experimental examples demonstrating the physiologic influence of temperature on size (Bizer, 1978; Stillwell & Fox, 2009), and many examples of recent body size change in wild populations associated with increased temperatures (Gardner et al., 2019; Weeks et al., 2020; Wonglersak et al., 2020), trends are not universal (Baar et al., 2018; Salewski et al., 2010; Sheridan & Bickford, 2011). This lack of uniform responses suggests that the negative impact of temperature on size is not acting alone or is not always the main factor dictating size as climate changes. For example, in temperate regions, warmer temperatures also bring longer growing seasons, which may act to increase body size (Chown & Klok, 2003; Davison & Field, 2017; Eastman et al., 2012). Additionally, changes in precipitation are expected to alter primary productivity and water availability, both of which can impact body size (Dubos et al., 2018; Kelly et al., 2018; Morales‐Castilla et al., 2012; Yom‐Tov & Geffen, 2006). Despite this, relatively few studies have examined how changes in precipitation, in conjunction with changes in temperature, have influenced body size compared to the number examining impacts of temperature on size. Examining these factors simultaneously presents an opportunity to test hypotheses regarding the mechanisms of observed size changes.

Two main hypotheses exist regarding the impact of precipitation on body size. First, for both endotherms and ectotherms, the resource hypothesis posits that individuals can grow larger in areas with more resources (Rosenzweig, 1968), so areas with higher primary productivity, often correlated with rainfall within a given area, should support larger individuals. Second, the desiccation‐resistance hypothesis posits that larger individuals are better able to resist desiccation through their smaller surface area:volume ratio, and thus individuals, particularly ectotherms, in dry environments are proposed to be larger to minimize water loss (Bujan et al., 2016; Nevo, 1973). Tests of the relationship between precipitation and body size over decades of climate change allow for explicit tests of these hypotheses and provide a powerful tool for improving our understanding of expected impacts of climate change on size.

Studies examining how changes in precipitation and temperature have impacted organism size over the past decades have found few consistent trends, and interactions between temperature and precipitation remain underexplored. We searched Web of Science for “body size” and “precipitation” in August 2021 and looked at papers examining how recent changes in precipitation affect body size. Slightly more species (*n* = 19) have been found to decline in size with increasing precipitation (birds: Onley et al., 2020; Weeks et al., 2020; beetles: Baar et al., 2018; Tseng et al., 2018) than to increase in size (*n* = 15; mammals: Martin & Barboza, 2020; birds: Campbell‐Tennant et al., 2015; Gardner et al., 2014; Onley et al., 2020; reptiles: Stanley et al., 2020; beetles: Baar et al., 2018; Tseng et al., 2018), but many species (*n* = 24) have shown no relationship between precipitation and size (mammals: Maldonado‐Chaparro et al., 2015; Rughetti & Festa‐Bianchet, 2012; birds: Aubry et al., 2013; Campbell‐Tennant et al., 2015; Dubos et al., 2019; Onley et al., 2020; reptiles: Stanley et al., 2020; beetles: Baar et al., 2018; Babin‐Fenske et al., 2008; wasps: Polidori et al., 2020). Four studies have reported testing for interactive effects between temperature and size, but approximately equal numbers have found evidence of interactions (birds: Bailey et al., 2020; Kruuk et al., 2015; amphibians: Caruso et al., 2014; Sheridan et al., 2018), as have found no evidence for an interaction (birds: Dubos et al., 2018; Gardner et al., 2019; reptiles: Stanley et al., 2020). Two of the four studies to date which report a significant interaction between temperature and precipitation show that at high temperatures, size is greater in dry conditions than in wet conditions (supporting the dessiccation resistance hypothesis), but at lower temperatures size is greater in wet conditions than in dry conditions (supporting the resource hypothesis; Kruuk et al., 2015; Sheridan et al., 2018). One study shows the opposite (Caruso et al., 2014) and another shows opposing trends across different size measures (Bailey et al., 2020).

Because climate changes do not involve temperature or precipitation changing in isolation, studies that explicitly examine multiple climate variables, and test for interactions between them, are necessary to improve predictions of how organisms will respond to ongoing climate change. We propose that interactions between temperature and precipitation may help explain the lack of consistent size declines with climate warming reported in the literature. Here, we examine 18 frog species collected from Borneo over 136 years and locally recorded temperature and precipitation. To date, only a small number of studies have directly examined how changes in climate relate to changes in amphibian body size. As with studies on other taxonomic groups, results have been mixed. Looking at long periods of evolutionary time, some studies have found that amphibians are larger in periods of low temperature and high precipitation, supporting the resource hypothesis outlined below (Martinez‐Monzon et al., 2018). Other studies have found similar support for the resource hypothesis but note that size is related to primary productivity (which is influenced by both temperature and precipitation), but not to temperature or precipitation directly (Martínez‐Monzón et al., 2022). Studies examining responses to more recent climate changes have had inconsistent results. For example, Tryjanowski et al. (2006) examined males and females of three species of *Rana* in Poland, and found that only half showed significant responses of size to North Atlantic Oscillation index, a measure of temperature and precipitation. Body condition of *Bufo bufo*, a common toad, decreased with increased temperature (Reading, 2007), but there was no relationship between body size and temperature for the toad *Anaxyrus fowleri* (Green & Middleton, 2013). Additional similar examples exist (Sheridan et al., 2018), emphasizing that to date, it is challenging to predict how amphibians will respond to future climate warming and altered precipitation.

To test how temperature, precipitation, and their interaction influence anuran body size, we use a generalized‐linear mixed‐effects modeling framework (GLMM) and explicitly test the hypotheses below regarding drivers of size changes.

Hypothesis 1 Climate (temperature and precipitation) has no impact on size.

If climate does not impact size, we would expect to find no relationship between size and temperature, precipitation, or their interaction.

Hypothesis 2 Temperature drives size.

If temperature is the main factor dictating size, we would expect smaller sizes at warmer temperatures due to the physiological principles outlined above (e.g., Bergmann's Rule and temperature‐size rule). Additionally, we would expect temperature to have a larger effect size than precipitation, and a lack of interaction between temperature and precipitation.

Hypothesis 3 Precipitation drives size.

If precipitation is the main factor dictating size, we would expect to see either (a) larger sizes under dry conditions due to the desiccation hypothesis, or (b) larger sizes under wet conditions due to the resource hypothesis. Additionally, we would expect precipitation to have a larger effect size than temperature, and a lack of interaction between precipitation and temperature.

Hypothesis 4 Temperature and precipitation interact to influence size.

If temperature and precipitation interact to influence body size, then we would expect a significant interactive effect demonstrating that the relationship between precipitation and body size changes as climates warm. Because amphibians are particularly vulnerable to desiccation due to their water‐permeable skin, we would expect larger individuals in warmer and drier environments due to their lower surface area:volume ratio, which helps prevent desiccation (Olalla‐Tarraga et al., 2009). Only one study examines interactions between temperature and precipitation on frog body size (Sheridan et al., 2018), and we seek to test this for other anurans.

## 2 MATERIALS AND METHODS

### 2.1 Anuran body size

We examined the majority of Borneo frog specimens held in museums around the world and selected for study those species which had a minimum of 20 individuals collected across at least 20 years, with a minimum of nine collection years. We excluded species with taxonomic uncertainty or those whose taxonomy has changed considerably since the 1880s, such as many members of the genus *Philautus* and *Meristogenys*, to avoid taxonomic error in assigning species. We also excluded species with extreme ranges of adult sizes observed in the wild, such as large‐bodied *Limnonectes* (88–175 mm) and *Phrynoides* (95–153 mm) to avoid potential size differences due to age influencing our results. We only measured sexually mature adults, determined by external sexual characters or by internal examination of gonads. All specimens were measured with digital calipers to the nearest 1 mm, and all measurements were performed by JAS to avoid inter‐individual measurement bias (Lee, 1982). Thus, our dataset contained snout‐vent length (SVL) measurements of 3009 anuran specimens from Borneo, collected over 136 years (1872–2008). Data included a total of 18 species and 29 species‐sex analysis units (males and females were treated as separate units for analysis because of sexual size dimorphism; data available from the Dryad Digital Repository: https://doi.org/10.5061/dryad.rbnzs7hfh). These species represent 11 genera and 4 families, including tree frogs (Rhacophoridae), highly aquatic species (*Staurois latopalmatus*, e.g.), human commensals (*Polypedates leucomystax*, e.g.), fossorial forest species (*Megophrys nasuta*), and direct developers (*Philautus*).

### 2.2 Climate data

We used temperature data from a weather station in Sandakan, a city in eastern Sabah, as this weather station had the longest time series of temperature data from North Borneo (http://berkeleyearth.lbl.gov/auto/Stations/TAVG/Text/156691‐TAVG‐Data.txt). Temperature data were available for 111 years between 1880 and 2010 (130 years). We used precipitation data from a weather station in Kuching, a city in Sarawak, as this weather station had the longest time series of precipitation from North Borneo (https://www1.ncdc.noaa.gov/pub/data/ghcn/daily/). Total annual precipitation data were available for 118 years between 1876 and 2010 (134 years). We confirmed that patterns of high and low rainfall (total annual precipitation) in Sandakan and Kuching were similar using linear regression for overlapping years of data (*F*_1,97_ = 3.30, *p* = .07). Because the Kuching precipitation dataset was significantly larger than the Sandakan dataset for years in which frogs were collected, we used the Kuching data to avoid losing nearly half of our size measurements. While temperatures and precipitation vary across Borneo, a relatively hot or dry year in one location is likely to be a relatively hot or dry year in other locations in Sabah, Sarawak, and Brunei. Additionally, because 99% of our specimens come from Sabah and Sarawak, and given the temporal span of collection, these weather stations provide the best representative measures for climate.

For climate analyses, we used monthly mean temperature and monthly total precipitation. For size analyses, we used annual mean temperature, calculated from monthly averages for years with at least 11 months of data, and total annual precipitation for years with 12 months of data.

### 2.3 Data analyses

We analyzed data using a generalized linear modeling framework (GLMM; Zuur et al., 2009). First, we tested for signs of climate change over time by creating GLMMs for mean monthly temperature and total monthly precipitation, separately, with year as a fixed effect and month as a random effect to account for seasonality. Temperature and precipitation were log_10_‐transformed to meet assumptions of normality.

**FIGURE 1:**
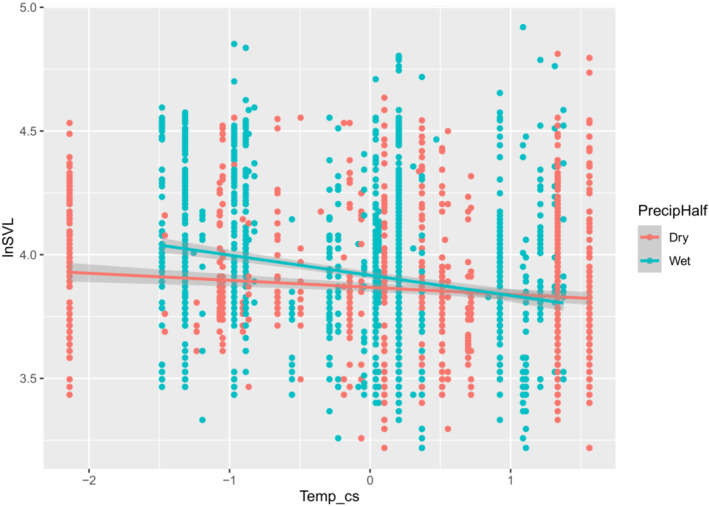
Body size (lnSVL) of Borneo frogs on scaled temperature for wet (blue) and dry (red) years with 95% conidence interval (gray bands).

**FIGURE 2:**
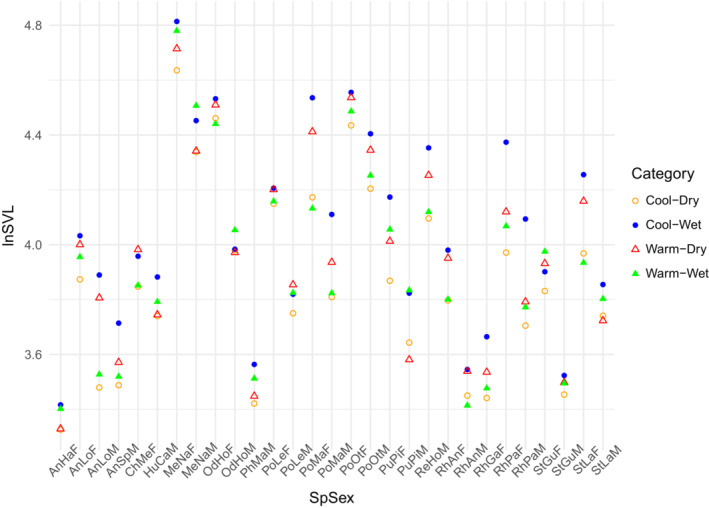
Fitted values for each species‐sex analysis unit, for four combinations of temperature and precipitation using the coolest and warmest temperatures in the dataset, and the lowest and highest precipitation values in the dataset. Full species names in Table S1.

We next examined long‐term trends in frog body size. We first examined body size irrespective of climate by modeling size (lnSVL) as a function of collection year, with random intercepts for the 29 species‐sex analysis units, and allowing slopes to vary for each species‐sex unit. Next, we created and compared various GLMMs that predicted frog body size using different combinations of an interaction between scaled mean annual temperature and scaled total annual precipitation, and collection year as fixed effects, with random intercepts for each species‐sex unit (Table 1). Temperature and precipitation were scaled in R using the “scale” function to create consistency across the dataset. Finally, we compared GLMMs with different fixed effects and model structures using Akaike information criterion (AIC) under maximum‐likelihood (ML). Results of our best model are presented with restricted maximum‐likelihood (REML). To visualize the interaction effect from the best model, we assigned each precipitation value the category of “dry” or “wet” based on whether it was in the bottom or top 50% of precipitation values, respectively. We then regressed body size (lnSVL) on scaled temperature for each category (Figure 1). Additionally, we calculated size of each species for cool‐dry, cool‐wet, warm‐dry, and warm‐wet years using the effect sizes for each species, and the coolest and the warmest temperature in the dataset, and the dryest and the wettest precipitation in the dataset (Figure 2).

**TABLE 1:** Akaike information criteria values for GLMMs predicting size (lnSVL) from temperature (Temp_cs) and precipitation (Precip_cs).

Model structure	df	ΔAIC
lmer(lnSVL~Temp_cs*Precip_cs + (1 + Temp_cs*Precip_cs|SpSex))	15	0.00
lmer(lnSVL~Temp_cs*Precip_cs + Year.Collected+(1 + Temp_cs + Precip_cs|SpSex))	12	18.54
lmer(lnSVL~Temp_cs*Precip_cs + (1|SpSex))	6	137.12
lmer(lnSVL~Temp_cs*Precip_cs + Year.Collected+(1|SpSex))	7	137.72

To allow for direct comparisons with other published studies that examine only temperature or precipitation, we also report the results of GLMMs with (a) temperature and (b) precipitation as fixed effects and species‐sex analysis units as a random effect (Table S2). All analyses were conducted in R version 4.0.3 (R Core Team, 2020). We used the *lme4* package. Relevant data and code to repeat analyses are in the Dryad Digital Repository: https://doi.org/10.5061/dryad.rbnzs7hfh).

## 3 RESULTS

### 3.1 Long‐term trends in temperature and precipitation

Our model of precipitation over time included 1480 monthly precipitation values from 1876 to 2010, and our model of temperature over time included 1330 monthly mean temperature values from 1880 to 2010. Precipitation increased over the study period (7.80 × 10^−4^, SE = 2.74 × 10^−4^, *p* = .004; see Table S3 for full model results) but temperature did not change significantly (2.53 × 10^−5^, SE = 1.46 × 10^−5^, *p* = .08; Table S3). Note that there was an increase in temperature from 1950 to 2010 (6.77 × 10^−4^, SE = 3.64 × 10^−5^, *p* < .001; see Table S3), in line with other published climate data (https://www.ncei.noaa.gov/access/monitoring/climate‐at‐a‐glance/global/time‐series).

### 3.2 Long‐term trends in body size

Our model contained body size (lnSVL) measures of 3009 specimens representing 18 frog species, collected over 138 years (1872–2010; Table S1). Seven species were only represented by one sex, resulting in 29 species‐sex units. Our model for size on year, accounting for differences across species‐sex units (SpSex as a random effect), showed that body size did not vary as a function of collection year (−2.48 × 10^−5^, SE = 6.73 × 10^−5^, *p* = .71; Table S3).

### 3.3 Changes in body size related to climate

Because of gaps in temperature and precipitation data (both temperature and precipitation were not available for every collection year), our dataset for comparison of body size and climate contained measurements of 2609 anuran specimens, spanning 117 years (1891–2008). The most competitive model from our analysis (Table 1) indicates that a crossover interaction between precipitation and temperature explains changes in body size (Table 2; see Tables S4 and S5 for fixed and random effects for each species, respectively). There were no noticeable differences in response for males and females (Tables S4 and S5).

**TABLE 2:** Fixed effect estimates from the best‐fit GLMM predicting 117 years of frog body size (lnSVL) data using scale‐transformed values for mean annual temperature (Temp_cs) and total precipitation (Precip_cs). Model is based on 2609 Bornean frog specimens representing 29 species‐sex units of 18 species. Species‐sex units were assigned random effects for slope and intercept. Model structure: Lmer(lnSVL~Temp_cs*Precip_cs + (1 + Temp_cs*Precip_cs**|**SpSex)).

	Estimate	Std. error	df	*t*‐value	*p*‐value
(Intercept)	3.954	0.0645	27.98	61.34	<.001
Temp_cs	−0.007	0.0037	24.56	−1.90	.069
Precip_cs	0.016	0.0032	24.45	4.91	<.001
Temp_cs:Precip_cs	−0.010	0.0029	18.43	−3.29	<.004

To visualize the crossover interaction, we assigned each precipitation value the category of “dry” or “wet,” based on whether it was in the bottom or top 50% of precipitation values, respectively. We then regressed body size (lnSVL) on scaled temperature for each category, showing that at cool temperatures, species were larger in wet years than in dry years, but that at warm temperatures, species were larger in dry years than in wet years (Figure 1).

## 4 DISCUSSION

Our data indicate that while species‐sex‐specific body size did not change over the sampling period (1872–2010), size was significantly related to the interaction between temperature and precipitation. We show that at cool temperatures, all frog species are smaller when it's dry than when it's wet, suggesting that the resource hypothesis is supported at cool temperatures (wetter = bigger; Figures 1 and 2). Interestingly, at warm temperatures, frogs are more likely to be smaller when it's wet, supporting the desiccation‐resistance hypothesis at warm temperatures (drier = bigger). The shift from resource influence on body size at cooler temperatures to desiccation resistance at warmer temperatures supports the principle that evaporative water loss increases at warmer temperatures. Thus, it is reasonable to expect that as the climate warms, anuran body sizes will shift from being driven by resource availability (cool and wet = larger) to desiccation resistance (warm and dry = larger). Our data provide evidence that rising temperatures alter the impact of precipitation on body size of Borneo frogs.

Amphibians may be particularly susceptible to the influence of precipitation on size compared to other taxa due to their permeable skin. While some studies have shown that increased rainfall leads to increased body size of birds and mammals (Gardner et al., 2014; Kruuk et al., 2015; Martin & Barboza, 2020; Onley et al., 2020), many more have shown that precipitation has no impact on body size of invertebrates, birds, and mammals (Baar et al., 2018; Gardner et al., 2019; Polidori et al., 2020; Salewski et al., 2014). Our models examining these variables in isolation show a similar trend for temperature, but an unexpected negative relationship between precipitation and size (Table S2). However, our full analyses reveal an interaction between temperature and precipitation, indicating in our case that while size is expected to decline in both wet and dry environments as temperatures warm, the magnitude of decline will be much greater in wet than in dry environments (Figure 1).

Studies on body size of any taxa, amphibian or otherwise, that include precipitation as well as temperature often report the influence of each variable independently, and interactive effects between temperature and precipitation have only been reported in a handful of studies (Bailey et al., 2020; Caruso et al., 2014; Gardner et al., 2016; Kruuk et al., 2015; Sheridan et al., 2018). As a result, it is difficult to know whether authors looked for such effects and did not find them, and thus did not report them, or whether they did not test for such interactions in their analyses. Some studies which specifically test interactions between temperature and precipitation show similar results to ours, in that size increases with temperature at low levels of precipitation, but declines at high levels of precipitation (Bailey et al., 2020; Kruuk et al., 2015). However, other studies showed the opposite pattern, with body condition (Gardner et al., 2016) or wing length (Bailey et al., 2020) increasing with temperature in wet environments and decreasing in dry environments. Further, Caruso et al. (2014) found the greatest size declines in warmer and drier environments, with smaller declines in warmer wetter environments, in direct contrast to our results. Our work demonstrates the need to simultaneously examine the impacts of changes in precipitation alongside temperature changes, and that independently testing the effects of temperature and precipitation may obscure important and more complex patterns in physiological responses to climate change.

Recent Intergovernmental Panel on Climate Change data indicate that many areas are expected to become wetter as the climate warms (IPCC, 2021). Thus, our data suggest that frogs and other amphibians may become smaller across most of Europe, Asia, North America, and Australia, with lesser size declines in parts of Southeast Asia, the Amazon basin, southern Africa, and Madagascar. We encourage additional examination of the interaction between temperature and precipitation on amphibian size, especially in tropical areas, to better predict broad responses to future changes in temperature and precipitation around the globe.

Body size declines resulting from changes in temperature and precipitation are likely to lead to increased thermal stress (Buckley, 2021; Peralta‐Maraver & Rezende, 2021), further exacerbating the negative impacts of body size declines on populations. Further, the maximum metabolic rate that organisms can sustain at stressful temperatures declines with the duration of exposure at a slightly faster rate for smaller organisms. Thus, increased duration and intensity of heatwaves expected from continued climate change are likely to lead to increased mortality of small‐bodied species compared to larger congeners, and of organisms that have become smaller due to decades of global warming. Because heatwaves are predicted to occur more frequently but may not be detected when analyzing temperature on an annual mean basis, heat stress may be more prevalent than an examination of mean temperature alone indicates (Perkins‐Kirkpatrick & Lewis, 2020; Vasseur et al., 2014). Thus, we recommend examining body size changes of organisms in areas known to have experienced substantial increases in heat waves such as northeast South America, particularly Suriname, French Guiana, and northeast Brazil; northeast Africa, particularly northern Algeria and Somalia; and the Arabian Peninsula (Perkins‐Kirkpatrick & Lewis, 2020).

The full consequences of body size declines due to climate change are not yet known, but are expected to include population declines due to the link between size and reproductive output for many species (Roff, 2002), increased thermal stress, and consequent increases in mortality (Buckley, 2021; Peralta‐Maraver & Rezende, 2021). For amphibians in particular, size declines can make individuals more susceptible to diseases such as chytrid (Carey et al., 2006), and result in higher energetic demands (Wu et al., 2018). Because body size declines are not universal, predicting which species or regions are likely to be most affected remains a challenge. However, our data provide strong evidence that rising temperatures alter the impact of precipitation on body size of Borneo frogs, suggesting that examining precipitation in conjunction with temperature may provide more precise answers to such questions. We recommend additional work on this topic to predict population and community‐level impacts of climate‐induced size declines.

## AUTHOR CONTRIBUTIONS

**Jennifer Ann Sheridan:** Conceptualization (lead); data curation (lead); formal analysis (equal); investigation (lead); methodology (lead); visualization (equal); writing – original draft (lead); writing – review and editing (lead). **Chase Mendenhall:** Data curation (equal); formal analysis (equal); visualization (equal); writing – original draft (equal); writing – review and editing (equal). **Paul Yambun:** Data curation (supporting); investigation (supporting); resources (equal); writing – original draft (supporting); writing – review and editing (supporting).

## CONFLICT OF INTEREST

All authors have declared that they have no competing interests related to the current study.

## Supporting information


Table S1.
Table S2.
Table S3
Table S4.
Table S5.

## Data Availability

Data are available from the Dryad Digital Repository: https://doi.org/10.5061/dryad.rbnzs7hfh.
