# Supplementary material for: Frog body size responses to precipitation shift from resource‐driven to desiccation‐resistant as temperatures warm
Source: Ecol Evol. 2022 Dec 12;12(12):e9589. doi: 10.1002/ece3.9589 (PMC9745258; doi:10.1002/ece3.9589)
Supplement: Supplementary file 1 — Table S1. Table S2. Table S3 Table S4. Table S5. [file ECE3-12-e9589-s001.docx]

**supporting Information**

Table S1. Summary of species used in present study.

| **Family** | **Genus** | **Species** | **Sex** | **SpSex** | **n** | **Mean SVL** | **Min SVL** | **Max SVL** | **Min**  **Year** | **Max**  **Year** | **Year**  **Span** |
| --- | --- | --- | --- | --- | --- | --- | --- | --- | --- | --- | --- |
| Bufonidae | *Ansonia* | *hanitschi* | Female | AnHaF | 83 | 29 | 25 | 34 | 1895 | 2006 | 111 |
| Bufonidae | *Ansonia* | *longidigita* | Female | AnLoF | 44 | 54 | 44 | 65 | 1894 | 2007 | 113 |
| Bufonidae | *Ansonia* | *longidigita* | Male | AnLoM | 202 | 40 | 33 | 46 | 1894 | 2008 | 114 |
| Bufonidae | *Ansonia* | *spinulifer* | Male | AnSpM | 36 | 36 | 28 | 40 | 1956 | 2007 | 51 |
| Bufonidae | *Rentapia* | *hosii* | Male | ReHoM | 147 | 68 | 56 | 77 | 1892 | 2006 | 114 |
| Megophryidae | *Megophrys* | *nasuta* | Female | MeNaF | 25 | 117 | 98 | 139 | 1907 | 2007 | 100 |
| Megophryidae | *Megophrys* | *nasuta* | Male | MeNaM | 49 | 85 | 67 | 98 | 1899 | 2007 | 108 |
| Ranidae | *Chalcorana* | *megalonesa* | Female | ChMeF | 47 | 50 | 40 | 65 | 1929 | 2007 | 78 |
| Ranidae | *Huia* | *cavitympanum* | Male | HuCaM | 78 | 44 | 38 | 49 | 1958 | 2008 | 50 |
| Ranidae | *Odorrana* | *hosii* | Female | OdHoF | 119 | 90 | 74 | 102 | 1891 | 2007 | 116 |
| Ranidae | *Odorrana* | *hosii* | Male | OdHoM | 233 | 55 | 45 | 63.5 | 1956 | 2007 | 51 |
| Ranidae | *Pulchrana* | *picturata* | Female | PuPiF | 61 | 56 | *45* | 64 | 1956 | 2007 | 51 |
| Ranidae | *Pulchrana* | *picturata* | Male | PuPiM | 164 | 41 | 34 | 49 | 1950 | 2007 | 57 |
| Ranidae | *Staurois* | *guttatus* | Female | StGuF | 170 | 50 | 43 | 60 | 1872 | 2007 | 135 |
| Ranidae | *Staurois* | *guttatus* | Male | StGuM | 222 | 33 | 28 | 36 | 1894 | 2007 | 113 |
| Ranidae | *Staurois* | *latopalmatus* | Female | StLaF | 72 | 59 | 50 | 71 | 1892 | 2007 | 115 |
| Ranidae | *Staurois* | *latopalmatus* | Male | StLaM | 133 | 44 | 37 | 55 | 1898 | 2008 | 110 |
| Rhacophoridae | *Philautus* | *macroscelis* | Male | PhMaM | 50 | 33 | 25 | 42 | 1987 | 2008 | 21 |
| Rhacophoridae | *Polypedates* | *leucomystax* | Female | PoLeF | 75 | 65 | 58 | 74 | 1894 | 2008 | 114 |
| Rhacophoridae | *Polypedates* | *leucomystax* | Male | PoLeM | 235 | 45 | 36 | 53 | 1894 | 2008 | 114 |
| Rhacophoridae | *Polypedates* | *macrotis* | Female | PoMaF | 91 | 75 | 57 | 90 | 1891 | 2007 | 116 |
| Rhacophoridae | *Polypedates* | *macrotis* | Male | PoMaM | 176 | 51 | 37 | 62 | 1929 | 2007 | 78 |
| Rhacophoridae | *Polypedates* | *otilophus* | Female | PoOtF | 49 | 91 | 79 | 97 | 1893 | 2007 | 114 |
| Rhacophoridae | *Polypedates* | *otilophus* | Male | PoOtM | 71 | 74 | 63 | 82 | 1893 | 2007 | 114 |
| Rhacophoridae | *Rhacophorus* | *angulirostris* | Female | RhAnF | 23 | 49 | 42 | 53.5 | 1892 | 2007 | 115 |
| Rhacophoridae | *Rhacophorus* | *angulirostris* | Male | RhAnM | 94 | 33 | 28 | 36 | 1891 | 2008 | 117 |
| Rhacophoridae | *Rhacophorus* | *gauni* | Female | RhGaF | 46 | 34 | 29 | 40 | 1963 | 1991 | 28 |
| Rhacophoridae | *Rhacophorus* | *pardalis* | Female | RhPaF | 30 | 65 | 54 | 81 | 1950 | 2007 | 57 |
| Rhacophoridae | *Rhacophorus* | *pardalis* | Male | RhPaM | 184 | 48 | 38 | 58 | 1950 | 2007 | 57 |

Table S2. Fixed effect estimates from the GLMM predicting frog body size (lnSVL). Models S1 and S2 are based on 2,676 total annual precipitation and body size values spanning 117 years (1891–2008). Models S3 and S4 are based on 2,939 mean annual temperature and body size values spanning 117 years (1891–2008).

Model S1: Precipitation effects on body size.

Estimate Std. Error df t-value p-value

(Intercept) 3.956 0.065 27.99 61.1 < 0.001

Precip_cs -0.017 0.001 2646 12.2 < 0.001

Model structure: lmer(lnSVL~Precip_cs+(1|SpSex))

Model S2: Precipitation effects on body size with variable slopes.

Estimate Std. Error df t-value p-value

(Intercept) 3.956 0.065 27.99 61.243 < 0.001

Precip_cs -0.016 0.004 27.22 4.314 < 0.001

Model structure: lmer(lnSVL~Precip_cs+(1+Precip_cs|SpSex))

Model S3: Temperature effects on body size.

Estimate Std. Error df t-value p-value

(Intercept) 3.956 0.065 27.99 60.926 < 0.001

Temp_cs -5.461 × 10^-3^ 1.407 × 10^-3^ 2909 -3.882 < 0.001

Model structure: lmer(lnSVL~Temp_cs+(1|SpSex))

Model S4: Temperature effects on body size with variable slopes.

Estimate Std. Error df t-value p-value

(Intercept) 3.956 0.065 27.99 61.192 < 0.001

Temp_cs -0.006 0.003 27.06 -2.054 0.0498

Model structure: lmer(lnSVL~Temp_cs+(1+Temp_cs|SpSex))

Table S3. Estimates of fixed effects from the GLMM quantifying changes in monthly mean temperature, monthly total precipitation, and size (lnSVL) of frogs in Borneo. Model 1 is based on 1,479 total monthly precipitation values spanning 134 years (1876–2010). Model 2 is based on 1,329 monthly mean temperature values spanning 130 years (1880–2010). Model 3 is based on 717 monthly mean temperature values spanning 59 years (1951–2010). Model 4 is based on 3,009 size measurements spanning 1872–2010. Temperature and precipitation were natural log-transformed to meet assumptions of linear modeling.

Model 1: Precipitation change over time.

Estimate Std. Error df t-value p-value

(Intercept) 4.161 0.544 1279 7.649 < 0.001

Year 7.798 × 10^-4^ 2.739 × 10^-4^ 1466 2.847 0.004

Model structure: lmer(lnPrecip~Year+(1|Month))

Model 2: Temperature change over time.

Estimate Std. Error df t-value p-value

(Intercept) 3.258 0.029 1239 112.450 < 0.001

Year 2.525 × 10^-5^ 1.462 × 10^-5^ 1316 1.727 0.084

Model structure: lmer(lnTemp~Year+(1|Month))

Model 3: Temperature change from 1951–2010.

Estimate Std. Error df t-value p-value

(Intercept) 1.963 0.072 710.3 27.19 < 0.001

Year 6.771 × 10^-4^ 3.636 × 10^-5^ 704.0 18.62 < 0.001

Model structure: lmer(lnTemp~Year+(1|Month))

Model 4: Body size change over time.

Estimate Std. Error df t-value p-value

(Intercept) 4.004 0.148 650.3 26.978 < 0.001

Year -2.478 × 10^-5^ 6.729 × 10^-5^ 2979 -0.368 0.713

Model structure: lmer(lnSVL~Year+(1|SpSex))

Table S4. Fixed effect estimates from the best-fit GLMM in Table 1 predicting 117 years of frog body size (SVL) data using scaled mean annual temperature (Temp_cs) and total annual precipitation (Precip_cs).

| Species:Sex unit | (Intercept) | Temp_cs | Precip_cs | Temp_cs:Precip_cs |
| --- | --- | --- | --- | --- |
| *Ansonia hanitschi:*  Female | 3.3744 | -0.0015 | 0.0167 | -0.0008 |
| *Ansonia longidigita:* Female | 3.9698 | 0.0020 | 0.0123 | -0.0089 |
| *Ansonia longidigita:* Male | 3.6817 | -0.0150 | 0.0156 | -0.0300 |
| *Ansonia spinulifer:* Male | 3.5809 | -0.0164 | 0.0187 | -0.0121 |
| *Chalcorana megalonesa:* Female | 3.9092 | -0.0008 | -0.0013 | -0.0105 |
| *Huia cavitympanum:* Male | 3.7978 | -0.0107 | 0.0197 | -0.0041 |
| *Megophrys nasuta:* Female | 4.7452 | 0.0029 | 0.0252 | -0.0049 |
| *Megophrys nasuta:* Male | 4.4199 | 0.0070 | 0.0283 | 0.0022 |
| *Odorrana hosii:* Female | 4.4864 | -0.0068 | 0.0006 | -0.0061 |
| *Odorrana hosii:* Male | 3.9998 | 0.0079 | 0.0085 | 0.0034 |
| *Philautus macroscelis:* Male | 3.4944 | -0.0039 | 0.0213 | -0.0034 |
| *Polypedates leucomystax:* Female | 4.1795 | -0.0012 | 0.0017 | -0.0043 |
| *Polypedates leucomystax:* Male | 3.8129 | 0.0101 | 0.0044 | -0.0043 |
| *Polypedates macrotis:* Female | 4.3185 | -0.0279 | 0.0106 | -0.0280 |
| *Polypedates macrotis:* Male | 3.9288 | -0.0238 | 0.0206 | -0.0180 |
| *Polypedates otilophus:* Female | 4.5060 | 0.0007 | 0.0077 | -0.0074 |
| *Polypedates otilophus:* Male | 4.3061 | -0.0060 | 0.0119 | -0.0127 |
| *Pulchrana picturata:* Female | 4.0411 | -0.0014 | 0.0363 | -0.0114 |
| *Pulchrana picturata:* Male | 3.7373 | -0.0044 | 0.0440 | 0.0032 |
| *Rentapia hosii:* Male | 4.2113 | -0.0145 | 0.0139 | -0.0170 |
| *Rhacophorus angulirostris:* Female | 3.8842 | -0.0082 | 0.0044 | -0.0145 |
| *Rhacophorus angulirostris:* Male | 3.4865 | -0.0081 | -0.0023 | -0.0096 |
| *Rhacophorus gauni:* Female | 3.5369 | -0.0146 | 0.0177 | -0.0123 |
| *Rhacophorus pardalis:*Female | 4.1483 | -0.0242 | 0.0372 | -0.0198 |
| *Rhacophorus pardalis:* Male | 3.8571 | -0.0317 | 0.0390 | -0.0178 |
| *Staurois guttatus:* Female | 3.9128 | 0.0182 | 0.0117 | -0.0012 |
| *Staurois guttatus:* Male | 3.4948 | 0.0005 | 0.0070 | -0.0032 |
| *Staurois latopalmatus:* Female | 4.0833 | -0.0222 | 0.0081 | -0.0223 |
| *Staurois latopalmatus:* Male | 3.7884 | -0.0080 | 0.0198 | -0.0015 |

Table S5. Random effect estimates from the best-fit GLMM in Table 1 predicting 117 years of frog body size (SVL) data using scaled mean annual temperature (Temp_cs) and total annual precipitation (Precip_cs).

| Species:Sex unit | (Intercept) | Temp_cs | Precip_cs | Temp_cs:Precip_cs |
| --- | --- | --- | --- | --- |
| *Ansonia hanitschi:*  Female | -0.5806 | 0.0055 | 0.0009 | 0.0088 |
| *Ansonia longidigita:* Female | 0.0148 | 0.0089 | -0.0036 | 0.0007 |
| *Ansonia longidigita:* Male | -0.2733 | -0.0080 | -0.0002 | -0.0204 |
| *Ansonia spinulifer:* Male | -0.3740 | -0.0094 | 0.0029 | -0.0026 |
| *Chalcorana megalonesa:* Female | -0.0457 | 0.0061 | -0.0171 | -0.0009 |
| *Huia cavitympanum:* Male | -0.1571 | -0.0037 | 0.0038 | 0.0054 |
| *Megophrys nasuta:* Female | 0.7903 | 0.0099 | 0.0094 | 0.0047 |
| *Megophrys nasuta:* Male | 0.4650 | 0.0140 | 0.0125 | 0.0118 |
| *Odorrana hosii:* Female | 0.5315 | 0.0001 | -0.0152 | 0.0035 |
| *Odorrana hosii:* Male | 0.0449 | 0.0148 | -0.0074 | 0.0130 |
| *Philautus macroscelis:* Male | -0.4606 | 0.0031 | 0.0055 | 0.0062 |
| *Polypedates leucomystax:* Female | 0.2245 | 0.0057 | -0.0141 | 0.0053 |
| *Polypedates leucomystax:* Male | -0.1420 | 0.0170 | -0.0115 | 0.0053 |
| *Polypedates macrotis:* Female | 0.3636 | -0.0210 | -0.0052 | -0.0184 |
| *Polypedates macrotis:* Male | -0.0262 | -0.0168 | 0.0048 | -0.0085 |
| *Polypedates otilophus:* Female | 0.5510 | 0.0076 | -0.0081 | 0.0022 |
| *Polypedates otilophus:* Male | 0.3512 | 0.0010 | -0.0039 | -0.0032 |
| *Pulchrana picturata:* Female | 0.0862 | 0.0055 | 0.0205 | -0.0018 |
| *Pulchrana picturata:* Male | -0.2177 | 0.0026 | 0.0281 | 0.0127 |
| *Rentapia hosii:* Male | 0.2563 | -0.0075 | -0.0020 | -0.0075 |
| *Rhacophorus angulirostris:* Female | -0.0708 | -0.0012 | -0.0114 | -0.0049 |
| *Rhacophorus angulirostris:* Male | -0.4685 | -0.0011 | -0.0181 | -0.0000 |
| *Rhacophorus gauni:* Female | -0.4181 | -0.0076 | 0.0019 | -0.0027 |
| *Rhacophorus pardalis:*Female | 0.1934 | -0.0172 | 0.0213 | -0.0102 |
| *Rhacophorus pardalis:* Male | -0.0978 | -0.0247 | 0.0232 | -0.0082 |
| *Staurois guttatus:* Female | -0.0422 | 0.0251 | -0.0042 | 0.0084 |
| *Staurois guttatus:* Male | -0.4601 | 0.0074 | -0.0088 | 0.0064 |
| *Staurois latopalmatus:* Female | 0.1284 | -0.0152 | -0.0078 | -0.0127 |
| *Staurois latopalmatus:* Male | -0.1665 | -0.0010 | 0.0040 | 0.0081 |
